# Fabrication, Structural Characteristics, and Properties of Sericin-Coated Wool Nonwoven Fabrics

**DOI:** 10.3390/ijms241914750

**Published:** 2023-09-29

**Authors:** Hye Gyeoung Lee, Mi Jin Jang, In Chul Um

**Affiliations:** 1 Department of Biofibers and Biomaterials Science, Kyungpook National University, Daegu 41566, Republic of Korea; 2 Preclinical Research Center, Daegu-Gyeongbuk Medical Innovation Foundation, Daegu 41061, Republic of Korea

**Keywords:** wool, sericin, nonwoven fabric, structural characteristics, mechanical properties, cell viability

## Abstract

Recently, nonwoven fabrics from natural silk have attracted considerable attention for biomedical and cosmetic applications because of their good mechanical properties and cytocompatibility. Although these fabrics can be easily fabricated using the binding character of sericin, the high cost of silk material may restrict its industrial use in certain areas. In this study, sericin was added as a binder to a cheaper material (wool) to prepare wool-based nonwoven fabrics and investigate the effect of the amount of sericin added on the structural characteristics and properties of the wool nonwoven fabric. It was found using SEM that sericin coated the surface of wool fibers and filled the space between them. With an increase in sericin addition, the porosity, moisture regain, and the contact angle of the sericin-coated wool nonwoven fabric decreased. The maximum stress and initial Young’s modulus of the nonwoven fabric increased with the increase in sericin amount up to 32.5%, and decreased with a further increase in the amount of sericin. Elongation at the end steadily decreased with the increase in sericin addition. All of the nonwoven fabrics showed good cytocompatibility, which increased with the amount of sericin added. These results indicate that sericin-coated wool-based nonwoven fabrics may be successfully prepared by adding sericin to wool fibers, and that the properties of these fabrics may be diversely controlled by altering the amount of sericin added, making them promising candidates for biomedical and cosmetic applications.

## 1. Introduction

Silk is composed of two biopolymers: fibroin and sericin. Sericin is removed from silk textiles to improve their luster and tactile feeling. Consequently, 50,000 tons of sericin are abandoned annually worldwide [1]. Recently, because of the discovery of its unique properties as a biomaterial, sericin has attracted considerable attention for biomedical, pharmaceutical, and cosmetic applications [2]. The advantageous properties of sericin include its biodegradability [3], antioxidant effect [4], good wound-healing effect [5], good water retention [6], excellent cell adhesion and growth [7], reduction in oxidative stress of liver cells [8], and good UV light-blocking properties [9].

Recently, natural silk nonwoven fabrics with good mechanical properties have been successfully fabricated using the binding character of sericin [10,11,12]. Sericin has a binding effect on fibers, which can be strengthened by wet treatment and hot pressing. Using this character of sericin, mass production of natural silk nonwoven fabrics is possible without the regeneration of silk, which results in a deterioration of its mechanical properties.

Silk/rayon nonwoven fabrics have also been fabricated to reduce the relatively high price of silk, and it was found that this composite fabric can be easily prepared using the same method as that for silk nonwoven fabrics, employing the binding effect of sericin [13].

Notably, a very small amount (2.6%) of sericin is required to bind rayon fibers for the fabrication of silk/rayon nonwoven fabric. This implies that only 2.6% sericin is needed to prepare nonwoven fabrics from fibers that have no binding character.

Wool is a cheaper fiber material than silk; it is used as a textile material with high water retention [14] and good heat preservance [15]. Wool is primarily composed of keratin (~95%) [16], which is used in hair and skin care cosmetics because of its smoothness, luster, softness, elasticity, and protective efficacy [17]. Furthermore, wool keratin has been studied for application in tissue engineering scaffolds [18]. Owing to the useful properties of wool, wool nonwoven fabrics have been prepared using different methods, including needle punching, stitch bonding for various applications including acoustic insulation [19], and geotextile [20]. Recently, Islam et al. prepared chitosan-added wool nonwoven fabric for wound dressing applications, and reported its good absorbency and excellent antimicrobial properties [21].

Previously, sericin has been added to wool to improve its dyeing and printability performance, increase its hydrophilicity [22], prevent its felting shrinkage [23], and endow it with antibacterial properties [24] and antistatic properties [25]. However, these studies were conducted to introduce the unique properties of sericin into wool textiles rather than make a new material using the binding character of sericin.

However, sericin may be used to prepare wool nonwoven fabric via hot pressing, using the binding effect of sericin. Furthermore, the good properties of sericin as a biomaterial can be introduced into wool. In the present study, various amounts of sericin were coated onto wool slivers to prepare a sericin-coated wool nonwoven fabric via wet treatment and hot pressing, and the effect of the amount of sericin on the structural characteristics and properties of the sericin-coated wool nonwoven fabric were examined.

## 2. Results and Discussion

### 2.1. Morphological Characteristics of the Sericin-Coated Wool Nonwoven Fabric

Table 1 shows photographs and field-emission scanning electron microscopy (SEM) images of sericin-coated wool nonwoven fabrics with different amounts of sericin added. In the case of wool without sericin (i.e., 0% sericin), because of the lack of a binder (sericin) for wool fibers, the wool fibers do not form a nonwoven fabric, displaying a bulky wool web. For 10.6–40.4% sericin addition, thin and compact nonwoven fabrics are formed, indicating that sericin effectively bound wool fibers to form nonwoven fabrics through wet and hot press treatments. These results are consistent with those of previous reports. Lee et al. reported that silk nonwoven fabric can be prepared at a sericin content of 8% [10]. Bae et al. reported that 2.6% sericin content was sufficient to form a silk/rayon nonwoven fabric [13]. Based on these results, it seems reasonable that wool-based nonwoven fabric can be fabricated at a sericin content of at least 10.6% and more, although we did not test below 10.6%.

Low-magnification SEM images show that the wool fibers in all nonwoven samples are arranged randomly, regardless of the amount of sericin, even though the wool fibers were processed using a carder to form a web. The wool fibers may be randomly oriented because wool fiber has a crimp (natural wave), and, despite carding, the wool fibers maintain their random arrangement in the web. At the same time, wet and hot press treatments may be responsible for the random orientation of wool fibers, considering that straight silk fibers become rugged after the wet and hot press treatments [11,13]. With increasing amounts of sericin added, more sericin on wool fibers and more sericin between wool fibers is observed, confirming that sericin was coated onto wool fibers and filled the space between the wool fibers. Moreover, with an increase in the amount of sericin added, the wool fibers adhered more to each other, with less pore space observed between the wool fibers. This is due to the binding effect of sericin on wool fibers. Sericin, swollen after the wet treatment, was deformed to bind wool fibers during hot pressing. This made the morphological structure of wool nonwoven fabric more compact. It has been previously reported that the silk web became more compact after hot pressing because of the binding effect of sericin [11,13].

High-magnification SEM images clearly show that sericin-coated wool fibers penetrated into the space between them, as indicated by the white circles in Table 1. At a sericin amount of 0%, scales on the wool fiber are observed. At 10.6%, some scales disappear because of the coating of sericin on wool fibers. At sericin amounts of 26.5% and above, sericin fills up the space between wool fibers, and the degree of filling increases with the amount of sericin.

**Figure 1:**
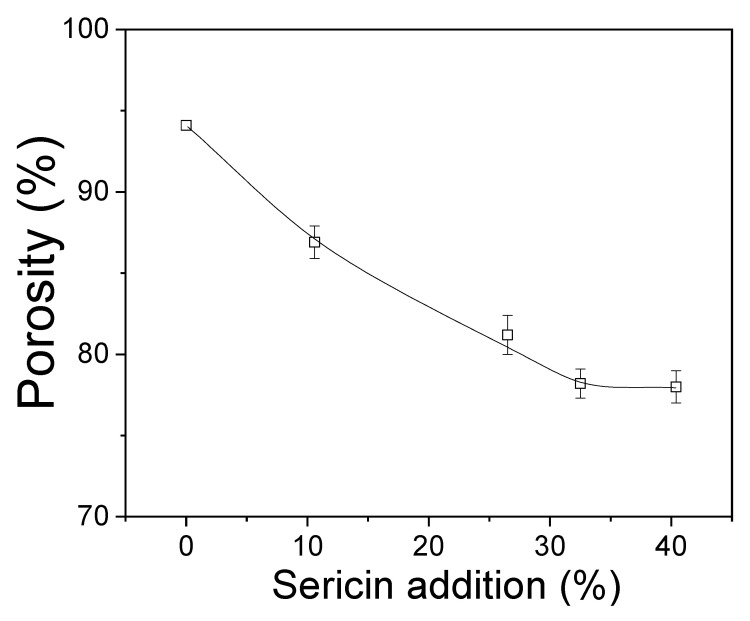
Porosity of sericin-coated wool nonwoven fabrics with different amounts of sericin (*n* = 3).

Figure 1 shows the porosities of sericin-coated wool nonwoven fabrics with different sericin amounts. In the case of 0% sericin, the nonwoven fabric exhibits a porosity of 94.1%. The porosity of the nonwoven fabric decreases to 78% with the increase in sericin amount to 32.5%. With further increases in the amount of sericin, the porosity does not change. The reduction in porosity occurred because of the compression of the nonwoven fabric during hot pressing. As the sericin amount increases, more sericin deforms and binds wool fibers. This reduces the void space between the wool fibers, leading to a decrease in porosity. Considering no further reduction in porosity after a sericin amount of 32.5%, this amount of sericin is the critical point for the maximum compression of sericin-coated wool nonwoven fabric. This result confirms the SEM results in Table 1.

### 2.2. Structural Characteristics of Sericin-Coated Wool Nonwoven Fabrics

**Figure 2:**
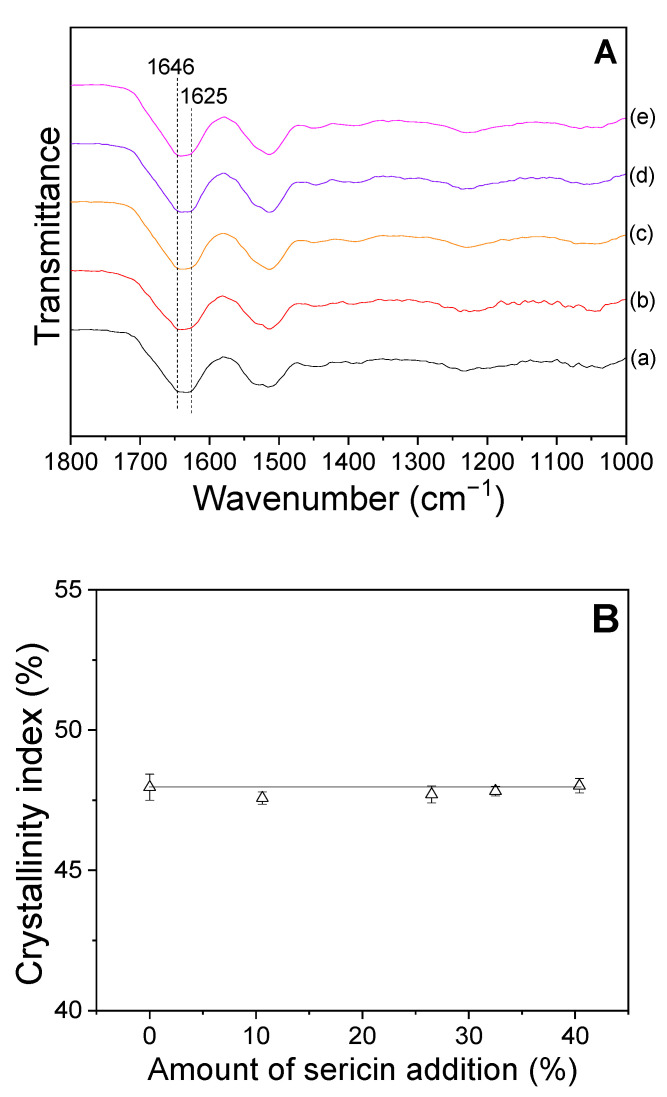
(**A**) Attenuated total reflection FTIR spectra and (**B**) crystallinity index of sericin-coated wool nonwoven fabrics with different amounts of sericin (*n* = 5); (a) 0%, (b) 10.6%, (c) 26.5%, (d) 32.5%, and (e) 40.4%.

Fourier transform infrared (FTIR) spectroscopy has been previously used to examine the molecular conformation and crystallinity of protein materials [26,27,28]. In the present study, FTIR measurements were performed on sericin-coated wool nonwoven fabrics to determine their crystallinity index (Figure 2). The sericin-coated wool nonwoven fabric shows IR absorption peaks at 1646 and 1625 cm^−1^. Sericin shows IR peaks at 1645 and 1620 cm^−1^, corresponding to random coil and β–sheet conformations, respectively [29]. Wool fiber exhibits IR peaks at 1620–1630 and near 1650 cm^−1^, which were attributed to β–sheet and α–helix conformations, respectively [16,30,31] Therefore, the IR absorption peak at 1646 cm^−1^ (Figure 2A) may be a result of the overlapping of two peaks corresponding to the random coils of sericin and the α–helix of wool. The IR peak at 1625 cm^−1^ was attributed to β–sheets of sericin and wool. More importantly, the FTIR spectra of sericin-coated wool nonwoven fabric do not change with changes in the amount of sericin. The crystallinity indices (Figure 2B) confirm this, exhibiting no changes with the amount of sericin added. Wool and sericin show very similar IR spectra. Therefore, it is difficult to determine the conformational changes in sericin-coated wool nonwoven fabrics with the change in the amount of sericin.

**Figure 3:**
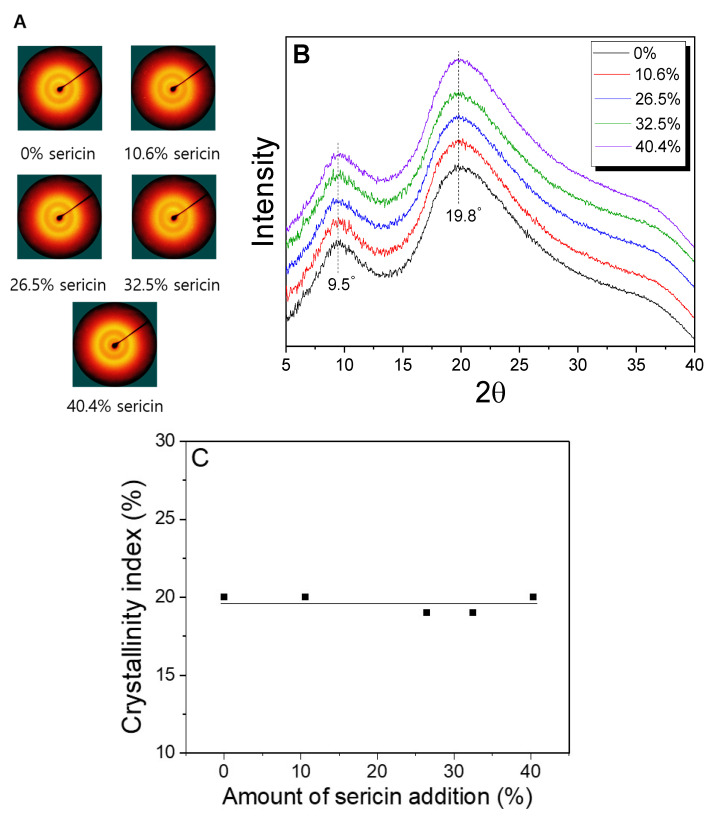
(**A**) X-ray diffraction patterns, (**B**) X-ray diffractograms, and (**C**) crystallinity index of sericin-coated wool nonwoven fabrics with different amounts of sericin.

Figure 3 shows X-ray diffraction (XRD) patterns and diffractograms of sericin-coated wool nonwoven fabric with different amounts of sericin added. All of the nonwoven fabrics exhibit two XRD rings corresponding to 2θ = 9.5° and 19.8°. The diffraction rings were attributed to randomly arranged wool fibers, confirming the SEM results (Table 1). The XRD patterns and diffractograms of the nonwoven fabric do not change with the amount of sericin. The diffraction peak at 9.5° was attributed to α-helix and β-sheet crystallites of wool keratin, and the peak at 19.8° was ascribed to β-sheet crystallites of wool keratin [16,32,33]. At the same time, a broad XRD peak at 19.2°, attributed to β–sheet crystallites, has been previously reported for sericin [34]. Unlike the FTIR spectrum, wool shows different X-ray diffraction peaks (at 9.5 and 19.8°) from sericin (at 19.8°). Nevertheless, X-ray diffractograms of nonwoven fabric do not change with the amount of sericin (Figure 3B). The results of the crystallinity index calculated from the diffractograms reconfirms that the overall crystallinity of the nonwoven fabric does not change (Figure 3C). This may be due to the weak crystallinity of sericin in the nonwoven fabric. It has been previously reported that β-sheet crystallites of sericin are disrupted by hot pressing at 200 °C [10]. Therefore, it seems that the low crystallinity of sericin does not affect the overall crystallinity of sericin-coated wool nonwoven fabrics with different amounts of sericin added.

**Figure 4:**
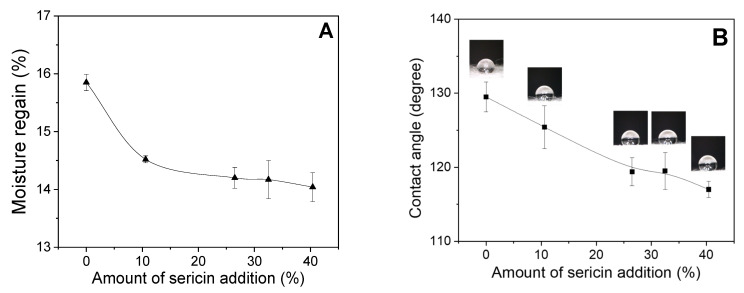
(**A**) Moisture regain (*n* = 3) and (**B**) contact angle (*n* = 10) of sericin-coated wool nonwoven fabrics with different amounts of sericin.

Figure 4 shows the moisture regain and contact angle of sericin-coated wool nonwoven fabric. With increasing sericin addition, the moisture regain and contact angle decrease. The decrease in moisture regain with increasing sericin content may be easily explained considering that the water absorption ability of wool is higher than that of silk. In other words, because the hydrophilicity of wool is higher than that of sericin, the overall hydrophilicity of nonwoven fabric decreases with the addition of sericin. The decrease in the contact angle of nonwoven fabric with sericin addition is consistent with previous reports that the hydrophilicity of wool increases by adding sericin [19]. This result is interesting, considering that the decrease in contact angle implies an increase in hydrophilicity. That is, the results of the moisture regain are opposite to those of the contact angle. The decrease in the contact angle of sericin-coated wool nonwoven fabric with increasing sericin amounts was attributed to the scales on the wool fiber. The surface of wool fibers has a scale, which is water-repellent. Consequently, the contact angle of the wool fiber is high (129.5°). However, with the addition of sericin to wool fiber, the water-repellent surface (scales) of the wool fibers is covered by hydrophilic sericin, and the space between the wool fibers is filled with sericin. This results in an increase in the hydrophilicity of the surface of the nonwoven fabric, leading to a decrease in the contact angle.

### 2.3. Mechanical Properties of the Sericin-Coated Wool Nonwoven Fabrics

**Figure 5:**
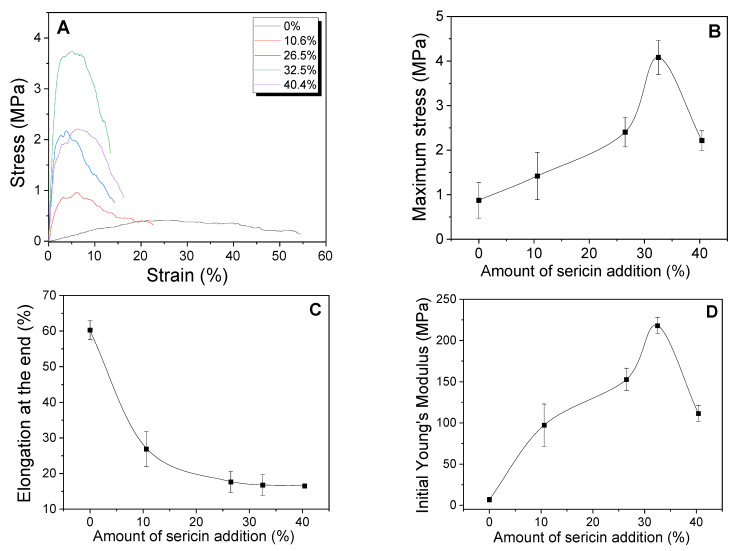
(**A**) Representative stress–strain curve, (**B**) maximum stress, (**C**) elongation at the end, and (**D**) initial Young’s modulus of the sericin-coated wool nonwoven fabric with different amounts of sericin (*n* = 5).

Figure 5 shows the mechanical properties of the sericin-coated wool nonwoven fabrics. The maximum stress and initial Young’s modulus of the nonwoven fabric increase with the increase in the amount of sericin up to 32.5%, and decrease afterward. At the same time, elongation at the end decreases with increasing amounts of sericin. These results indicate that a ductile character of the nonwoven fabric with low amounts of sericin (i.e., 0% and 10.6% sericin) changes to a stiff character at high amounts of sericin (32.5% and 40.4%). This transition was attributed to the binding effect of sericin on the nonwoven fabric. As the added amount of sericin increases, sericin binds more wool fibers, resulting in increases in strength and the Young’s modulus, as well as a decrease in elongation. Additionally, the morphological structure of the nonwoven fabric becomes denser and more compact with increasing sericin additions, leading to increases in the maximum stress and Young’s modulus.

Interestingly, the maximum stress of the nonwoven fabric decreases as the sericin addition exceeds 32.5%. This may be due to the excess of sericin in the wool nonwoven fabric. When sericin is added to wool until a certain amount, it acts as a binder for wool fibers. However, above the critical point, the excess sericin does not act as a binder, and does not contribute to the strength of the nonwoven fabric. Excess sericin may aggregate without binding wool fibers, decreasing the strength of the nonwoven fabric.

These results indicate that the maximum stress of wool nonwoven fabric could be increased significantly by adding sericin. A comparison with the results of previous reports confirms this. Santos et al., reported that wool felt with 672 g/m^2^ of sericin showed a tensile strength of 4.3 MPa [35], and Silva et al. reported that wool felt with 687 g/m^2^ had a tensile strength of 4.22 MPa [36]. Therefore, considering that sericin-coated wool nonwoven fabric with 100 g/m^2^ (i.e., it has much looser structure than those in the previous reports) showed a maximum stress of 4.08 MPa (32.5% sericin), this new preparation method of wool nonwoven fabric using the binding character of sericin can be an effective way to remarkably improve the strength of wool nonwoven fabric.

### 2.4. Effect of Sericin-Coated Wool Nonwoven Fabrics on Cell Viability

**Figure 6:**
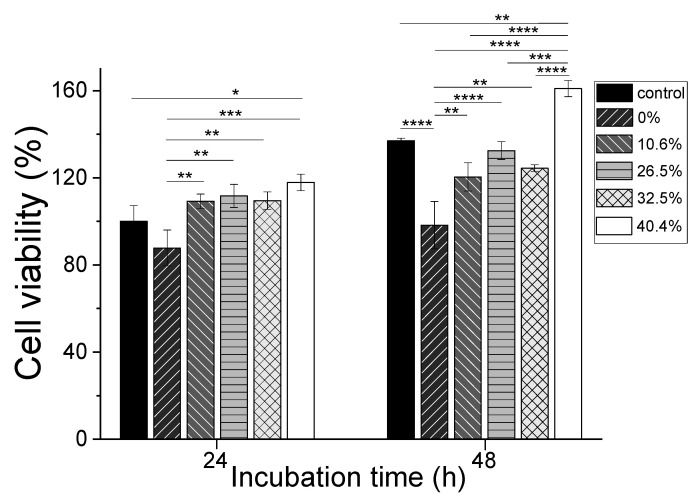
Effect of sericin-coated wool nonwoven fabrics on cell viability. Different amounts of sericin were added into the wool (* *p* < 0.05, ** *p* < 0.01, *** *p* < 0.001, **** *p* < 0.0001).

Figure 6 shows the effect of sericin-coated wool nonwoven fabrics on cell viability. Sericin-coated wool nonwoven fabrics (10.6–40.4% sericin) results in significantly higher (by 10.6%, 26.5%, and 32.5%, ** *p* < 0.01; 40.4%, *** *p* < 0.001) cell viability after 24 h of incubation than that of the wool nonwoven fabric without sericin (0% sericin). At an incubation time of 48 h, the difference between the nonwoven samples is greater and more significant. Wool nonwoven fabric (0% sericin) results in significantly (**** *p* < 0.0001) lower cell viability than the control. At the same time, all of the sericin-coated wool nonwoven fabrics (10.6–40.4% sericin) lead to significantly higher (by 10.6% and 32.5%, ** *p* < 0.01; 26.5% and 40.4%, **** *p* < 0.0001) cell viabilities than that of the wool nonwoven fabric (0% sericin). In particular, the wool nonwoven fabric with 40.4% sericin results in significantly different (by 10.6% and 32.5%, **** *p* < 0.0001; 26.5%, *** *p* < 0.001) cell viability from those of other sericin-coated wool nonwoven fabrics.

According to ISO 10993-5, materials resulting in cell viabilities of 80% or higher are considered nontoxic [37,38]. All of the nonwoven fabrics (0%–40.4% sericin) investigated in this study resulted in cell viabilities greater than 87.8%, indicating that these nonwoven fabrics can be considered nontoxic. This result is consistent with the positive effects of sericin [39,40,41,42] and wool [43,44,45] on cell viability. Additionally, significant increases in cell viability by nonwoven fabrics with the addition of sericin indicate that sericin results in better cell cytocompatibility than wool, and that the addition of sericin can be used to enhance the cytocompatibility of wool.

**Figure 7:**
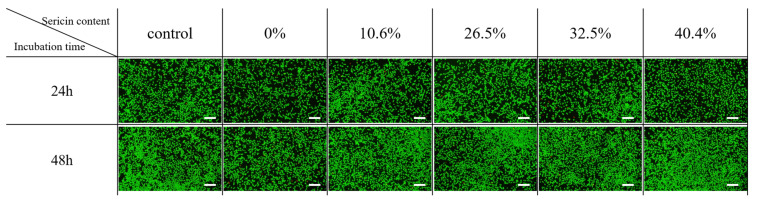
Fluorescence images of cell viability assays of sericin-coated wool nonwoven fabrics with different amounts of sericin. The scale magnification bars in the images represent 200 μm.

Figure 7 shows the positive effects of all of the nonwoven fabrics on cell viability, regardless of the added amount of sericin. More live cells are observed for sericin-coated wool nonwoven fabrics (10.6%–40.4% sericin) than for wool nonwoven fabrics (0% sericin). These results confirm the results of CCK-8 assays (Figure 6).

## 3. Materials and Methods

### 3.1. Materials

*Bombyx mori* Baekokjam silk cocoons were obtained from the National Institute of Agricultural Science (Wanju, Republic of Korea). Dichlorodicyanuric acid-treated wool slivers to partially remove the scales from the wool fibers were kindly provided by Cheil Industries Co (Gumi, Republic of Korea). Its fiber diameter was 17.4 µm.

### 3.2. Preparation of Sericin-Coated Wool Nonwoven Fabrics

**Figure 8:**
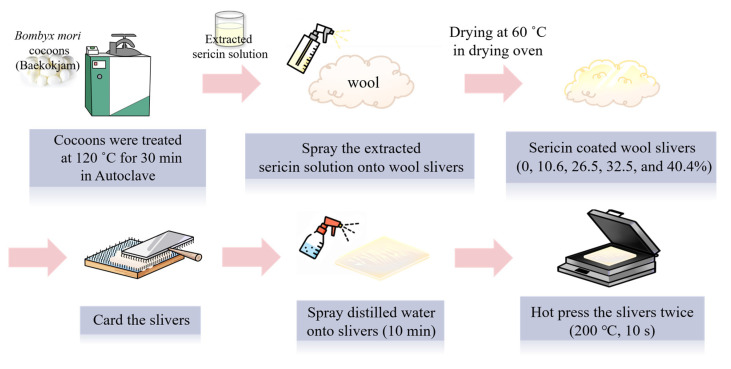
Schematic of the preparation of sericin-coated wool nonwoven fabrics.

The preparation procedure of sericin-coated wool nonwoven fabrics is shown in Figure 8. First, to extract silk sericin, the silkworm cocoons were immersed in purified water and treated at 120 °C for 30 min in an autoclave (JSAC-60, JSR, Gongju, Republic of Korea) [34]. The purified water was obtained using a reverse osmosis water purification system (RO50, Hana Science, Seongnam, Republic of Korea). The ratio of cocoons to purified water was set to 1:25. After extraction, the obtained sericin aqueous solution was filtered through the polyester nonwoven fabric (mass/area: 80 g/m^2^, thickness: 0.32 mm, permeability: 400 ccs, tensile strength: 18 kgf/5 cm) to prepare 1% (*w*/*w*) aqueous sericin solution. After that, different amounts of the extracted sericin solution were sprayed on wool slivers using a sprayer, and the wool slivers were dried at 60 °C in a drying oven (WOF-50, Daihan Scientific, Wonju, Republic of Korea). The dried sericin-coated wool slivers were carded using a lab-scale hand carder and blending board (Standard hand Cards, Brother drum carder, Silverton, CO, USA). The added amount of sericin was calculated using Equation (1), as follows:

(1) $$Added\;amount\;of\;sericin(\%)=\frac{{W_{1}-W}_{2}}{W_{2}}\times 100$$

where W_1_ is the dry weight of sericin-coated wool sliver after carding, and W_2_ is the dry weight of untreated wool slivers.

Wool slivers with different amounts of sericin added (0, 10.6, 26.5, 32.5, and 40.4 weight %) were prepared. One gram of wool slivers was used for the preparation of each 10 cm × 10 cm piece of sericin-coated wool nonwoven fabric. The sericin-coated wool slivers were then sprayed with distilled water for 10 min, and pressed twice using a hot press (HK 2008-1-5, Hankuk Industry Co., Gwangju, Republic of Korea) at 200 °C for 10 s to fabricate the sericin-coated wool nonwoven fabric. To prevent the adhesion of sericin-coated wool slivers to the plates of the hot press, polyester nonwoven fabrics were placed on the top and bottom of the sericin-coated wool slivers during the wet and hot press treatment. After the hot pressing, sericin-coated wool nonwoven fabric was obtained by removing the polyester nonwoven fabric.

### 3.3. Measurement and Characterization

The photos of sericin-coated wool nonwoven fabrics were obtained using a digital camera (Galaxy Note 20 Ultra, Samsung Inc., Suwon, Republic of Korea). The morphology of sericin-coated wool nonwoven fabrics was evaluated using field-emission SEM (S-4800, Hitachi, Tokyo, Japan) after coating with Pt–Pd.

The molecular conformation and crystallinity of sericin-coated wool nonwoven fabrics were examined using FTIR spectroscopy (Nicolet 380, Thermo Fisher Scientific, Waltham, MA, USA) with an attenuated total reflection (Smart iTR ZnSe) method. The scan range, scan number, and resolution were 4000–650 ${cm}^{-1}$, 32, and 8 ${cm}^{-1}$, respectively.

The crystallinity index was calculated from the intensity ratio of the peaks appearing at 1625 and 1646 ${cm}^{-1}$ in the FTIR spectrum using Equation (2) [29]. The FTIR measurements were performed seven times. The mean and standard deviation of the crystallinity index were calculated based on the five values remaining after the removal of maximum and minimum values.

(2) $$Crystallinity\;index(\%)=\frac{A_{1625{cm}^{-1}}}{A_{1625{cm}^{-1}}+A_{1646{cm}^{-1}}}\times 100$$

A_1625cm_^−1^ is the absorbance at 1625 ${cm}^{-1}$, and A_1646cm_^−1^ is the absorbance at 1646 ${cm}^{-1}$.

To determine the porosity of sericin-coated wool nonwoven fabrics, they were immersed in ethanol of volume $V_{1}$ for 5 min. After the nonwoven fabric sample was completely soaked in ethanol, the total volume ($V_{2}$) of ethanol and the sample was measured. The nonwoven fabric sample was then extracted from ethanol, and the residual volume of ethanol ($V_{3}$) was recorded. The porosity of the samples was then calculated using Equation (3) [46]. This method was used to determine porosity three times for each condition, and the average porosity of the samples was reported.

(3) $$Porosity\left(\%\right)=\frac{V_{1}-V_{3}}{V_{2}-V_{3}}\times 100$$

The crystalline structure and crystallinity of sericin-coated wool nonwoven fabrics were determined via wide-angle X-ray scattering. XRD patterns were obtained using a VANTEC500 (D8 Discover, Bruker, Karlsruhe, Germany) under Cu Kα radiation at 50 kV and 1000 μA for 600 s [11]. The XRD crystallinity index of the sericin-coated wool nonwoven fabrics was calculated using Equation (4) [47,48], as follows:

(4) $$XRD\;Crystallinity\;index\left(\%\right)=\frac{I_{9}-I_{14}}{I_{9}}\times 100$$

where I_9_ is the peak intensity at 2θ = 9°, and I_14_ is the peak intensity at 2θ = 14°.

To determine the moisture regain of the sericin-coated wool nonwoven fabrics, they were kept under standard conditions (20 °C and 65% relative humidity (RH)) for 24 h, and the moisture regain was calculated using Equation (5) [12]. The dry weight of the nonwoven fabric samples was determined using a moisture balance instrument (XM60, Precisa Gravimetrics, Dietikon, Switzerland).

(5) $$Moisture\;regain\left(\%\right)=\frac{Initial\;weight-Dry\;weight}{Dry\;weight}\times 100$$

The water contact angle was measured using a contact angle meter (Dino-Lite, AM703MZT, Seoul, Republic of Korea) following a sessile drop method using a 10 μL water droplet. The sericin-coated wool nonwoven fabric samples were cut into 2 cm × 2 cm pieces, and 10 μL of distilled water was placed on each sample at room temperature. The snapshots were made 30 s after the addition of the water drop, and the contact angle was calculated using these snapshots.

The mechanical properties of the sericin-coated wool nonwoven fabrics were measured using a universal testing machine (OTT-003, Oriental TM, Ansan, Republic of Korea). The tests were conducted using a 20 kgf load cell at an extension rate of 50 mm/min and a gauge length of 30 mm. The samples were cut into 50 mm × 10 mm pieces and preconditioned at 20 °C and 65% RH for 24 h. Seven samples were tested for each fabric specimen, and the average and standard deviation of the measurement results were calculated from the five results obtained by removing the maximum and minimum values.

L929 cells were grown in RPMI1640 medium (Gibco) supplemented with 10% (*v*/*v*) fetal bovine serum and 1% (*v*/*v*) antibiotic-antimytotic solution. The L929 cells were incubated at 37 °C in a humidified 5% CO_2_ atmosphere. When 80% confluence was observed, subcultures were performed twice per week.

The in vitro cytotoxicity tests of the fabrics were performed following a method specified in ISO 10993-5. Before extraction, each sample was sterilized with E.O. gas. The extraction was performed by immersing the samples (6 × 3 cm^2^) in the RPMI1640 culture medium (6 mL) with gentle shaking at 37 °C for 24 h. The ratio of the sample surface area to the extraction vehicle volume was 3 cm^2^/mL.

The cytotoxicity of the samples on L929 cells was determined by applying the CCK-8 (Cell Counting Kit 8, Dojindo, Japan) assay in vitro. The L929 cells were seeded into 96-well plates at 1 × 10^4^ cells/well and incubated at 37 °C for 24 h in a 5% CO_2_ atmosphere. The culture medium was then replaced with 100 µL/well of sample extracts. After 24 and 48 h of incubation, the extracts were discarded for the CCK assay, and 100 µL of a 10% (*v*/*v*) CCK-8 solution was added to each well. After 1 h of incubation, the absorbance was measured at 450 nm. Subsequently, the cell viability was calculated using Equation (6) [38].

(6) $$Cell\;viability(\%)=\frac{{OD}_{exp}-{OD}_{blank}}{{OD}_{control}-{OD}_{blank}}\times 100$$

The cytotoxicity was evaluated by performing fluorescence staining using a live/dead viability/cytotoxicity kit (L3224, Invitrogen, Waltham, MA, USA) following the manufacturer’s protocol. L929 cells were seeded into 24-well plates at 3 × 10^4^ cells/well and incubated for 24 h in a 5% CO_2_ atmosphere at 37 °C. Then, the culture medium was replaced with 300 µL/well of sample extracts. After 24 and 48 h of incubation, the extracts were discarded, and 300 µL of a staining solution was added to each well. After 45 min of incubation, the staining solutions were removed, and the cells were observed using a fluorescence inverted microscope (IX83, Olympus, Tokyo, Japan).

## 4. Conclusions

In this study, sericin-coated wool nonwoven fabrics were prepared using the binding character of sericin through a wet and hot press treatment, and the effect of the amount of sericin on the structural characteristics and properties of sericin-coated wool nonwoven fabrics was examined. The added sericin coated the surface of wool fibers and filled the space between wool fibers. As the amount of sericin increased, the porosity, moisture regain, and contact angle of the nonwoven fabrics decreased. The FTIR spectra and XRD results did not change with the addition of sericin. The maximum stress and initial Young’s modulus of the nonwoven fabrics increased with the increase in sericin addition up to 32.5%, and decreased for greater sericin amounts. Elongation at the end decreased with increasing sericin. Although all of the nonwoven fabrics resulted in good cell viability, the increase in sericin addition improved the cytocompatibility of the wool nonwoven fabrics. Therefore, wool-based nonwoven fabrics can be successfully fabricated using sericin, and various properties of these fabrics may be controlled via the amount of sericin added. These findings can be used as basic information for applied studies on wool-based nonwoven fabrics. Furthermore, to apply these nonwoven fabrics in bio-related fields including wound dressings and mask packs, it is thought that in vivo tests and cosmetic performance tests should be conducted as subsequent studies in the future.

## Figures and Tables

**Table 1:** Photographs and SEM images of sericin-coated wool nonwoven fabric with different amounts of added sericin. White scale bars represent 1.0 cm (photographs), 1.0 mm (SEM, low magnification), and 100 μm (SEM, high magnification).

	Image Type	Photograph	SEM Image (Low Magnification)	SEM Image (High Magnification)
Amount of Added Sericin				
0%		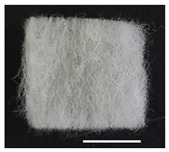	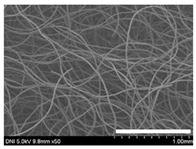	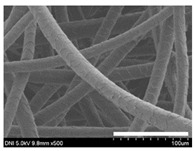
10.6%		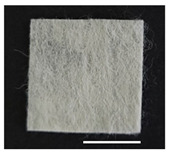	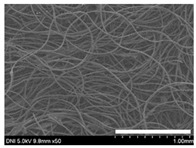	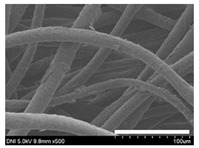
26.5%		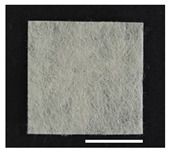	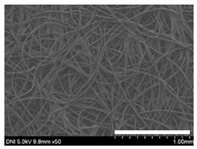	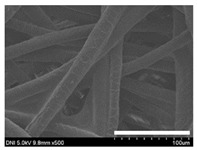
32.5%		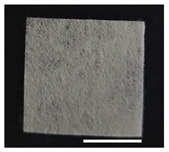	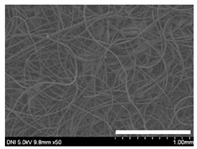	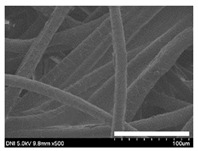
40.4%		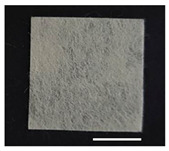	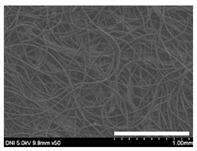	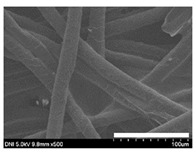

## Data Availability

The data presented in this study are available on request from the corresponding author.
